# From haploid to reference: a new milestone in poplar genomics

**DOI:** 10.48130/forres-0024-0018

**Published:** 2024-05-23

**Authors:** Shihui Niu, Quanzi Li

**Affiliations:** 1 State Key Laboratory of Efficient Production of Forest Resources, National Engineering Research Center of Tree Breeding and Ecological Restoration, College of Biological Sciences and Technology, Beijing Forestry University, Beijing 100083, PR China; 2 State Key Laboratory of Tree Genetics and Breeding, Chinese Academy of Forestry, Beijing 100091, PR China

**Keywords:** Haploid, Reference, Milestone, Poplars, Genomics

As the first tree species to undergo whole-genome sequencing, the completion of the *Populus trichocarpa* (black cottonwood) genome sequencing in 2006 marked a significant breakthrough in forest genomics research^[1]^. It greatly facilitates the adoption of poplar trees as a model species for genomics in the realm of forest trees^[2]^. The reference genome, serving as the bedrock of research plays an indispensable role in advancing genetic improvement of trees, research on adaptability, and ecosystem management. It enables scientists to more accurately pinpoint the genes that control important traits, thereby achieving more efficient variety breeding and resource utilization in forestry practice.

Genomics has advanced at an unprecedented rate in the past three years, especially in the technologies and strategies for genome assembly. For instance, the use of long-read sequencing technologies such as PacBio and Nanopore, in conjunction with high-throughput chromosome conformation capture (Hi-C) technology, has significantly improved the continuity and accuracy of genome assembly. To date, at least 3,517 genome datasets from 1,575 plant species have been released, 67% of which were completed in the past three years, including 73% of the newly published genomes that have been assembled to the chromosomal level^[3]^. Furthermore, the pan-genome studies have been introduced into forestry research, allowing researchers to explore the diversity and complexity within species' genomes^[4]^. Nonetheless, due to high heterozygosity and the presence of complex genomic regions, including centromeres and ribosomal RNA gene clusters (rDNA regions), the assembly of a truly complete genome is still a challenge^[5]^, even for model plants with smaller genomes like *Arabidopsis thaliana*^[6]^. The current v4.1version of the *P. trichocarpa* genome, as the highest quality among all the published poplar genomes, there are still 59 unresolved gaps which account for about ~3.32% of the genome. The ultimate goal of the T2T genome assembly is to achieve a completely gapless genome sequence, which is crucial for revealing the full biological information of the genome^[7]^.

The haploid materials have the characteristics of genotypic homozygous, which can obtain high-quality genome sequencing results, and have important application value in genetic engineering research^[8]^. A recent study by Liu et al., titled 'A nearly gapless, highly contiguous reference genome for a doubled haploid line of *Populus ussuriensis*, enabling advanced genomic studies'^[7]^, has successfully induced a double haploid (DH) callus line, referred to as DH15, from anthers of *P. ussuriensis*^[9]^. Utilizing this DH line, the authors have achieved a telomere-to-telomere (T2T) assembly of 19 chromosomes in the DH15 genome. Notably, this study has identified and annotated the centromere regions of the DH genome, marking a significant milestone as it reveals the content of the centromere complex regions within a poplar genome for the first time. This study has annotated 465 more genes than the annotation of *P. trichocarpa* genome. These advancements render a valuable resource for various studies on poplar genomes. In addition, the quality of the genome assembly has been greatly improved, from both the length of N50 and the number of gap sites, and the improved integrity of the genome significantly fills gaps present in the *P. trichocarpa* genome.

The availability of the T2T *P. ussuriensis* reference genome not only establishes a solid foundation for a deeper understanding of genomic structure and functions in poplar but also provides valuable resources for poplar genomic and evolutionary studies. In particular, the in-depth annotation of centromeric regions offers new insights into the mechanisms of chromosome distribution and cell division in plants. Furthermore, comparative analysis with other poplar genomes has revealed gene family expansion and contraction within the *Populus* genus, as well as the potential role of specific gene families in adaptive traits. These findings are crucial for understanding the biological characteristics of poplar trees. Through comparative genomics, researchers can identify gene families that are unique or expanded in poplars, which may be associated with the adaptability of poplars to environmental changes. This provides targets for further functional research and molecular breeding.

Although most gaps are nearly closed, a few remaining ones consist of the rDNA region, which bears the 18S-5.8S-25S ribosomal RNA genes also called nucleolus organizer regions (NORs) or 45S rDNA sites, presenting challenges for assembly due to their repetitive nature, copy number variation, and sequence complexity, which requires resolution in future research endeavors.

## Author contributions

Shihui Niu and Quanzi Li contributed equally for original draft and review the manuscript.

## Data availability

Data sharing not applicable to this article as no datasets were generated or analyzed during the current study.

## References

[1] (2006). The genome of black cottonwood, *Populus trichocarpa* (Torr. & Gray). Science.

[2] (2002). Genomics and forest biology: *Populus* emerges as the perennial favorite. The Plant Cell.

[3] (2024). Technology-enabled great leap in deciphering plant genomes. Nature Plants.

[4] (2024). The super-pangenome of *Populus* unveils genomic facets for its adaptation and diversification in widespread forest trees. Molecular Plant.

[5] (2024). Scalable telomere-to-telomere assembly for diploid and polyploid genomes with double graph. Nature Methods.

[6] (2022). A near-complete assembly of an *Arabidopsis thaliana* genome. Molecular Plant.

[7] (2024). A nearly gapless, highly contiguous reference genome for a doubled haploid line of *Populus ussuriensis*, enabling advanced genomic studies. Forestry Research.

[8] (2020). Puzzling out plant reproduction by haploid induction for innovations in plant breeding. Nature Plants.

[9] (2023). Exceptionally high genetic variance of the doubled haploid (DH) population of poplar. Journal of Forestry Research.

